# Can co-designed educational interventions help consumers think critically about asking ChatGPT health questions? Results from a randomised-controlled trial

**DOI:** 10.1038/s41746-025-02056-5

**Published:** 2025-11-17

**Authors:** Julie Ayre, Melody Taba, Brooke Nickel, Geoffrey Edlund, Trang Vu, Julia Yan, Lorna Butters, Ivan C. K. Ma, Kirsten J. McCaffery

**Affiliations:** 1 Sydney Health Literacy Lab, Sydney School of Public Health, Faculty of Medicine and Health, The University of Sydney, Sydney, NSW Australia; 2 Co-SHeLL, Sydney School of Public Health, Faculty of Medicine and Health, The University of Sydney, Sydney, NSW Australia

**Keywords:** Human behaviour, Public health

## Abstract

This randomised controlled trial evaluated two brief co-designed health literacy educational interventions (animation; images) to help people critically reflect on asking ChatGPT health questions. Australian adults with experience of ChatGPT, and without university education, were recruited via an online panel. Primary outcomes were intention to ask ChatGPT questions in ‘lower’ and ‘higher’ risk health scenarios. The analysis sample comprised 592 of 619 participants. The animation group (n = 191) reported lower intention to use ChatGPT for higher risk scenarios (M = 2.42/5, 95%CI: 2.27 to 2.56) compared to the images group (n = 203, M = 2.69/5, 95%CI: 2.54 to 2.83, p = 0.010). Both reported lower intentions compared to a control group who had not viewed the educational content (n = 205, M = 3.12/5, 95%CI: 2.98 to 3.27, p < 0.001). There was no effect on intentions to use ChatGPT for lower risk scenarios (p = 0.800). This study represents an initial step towards addressing health literacy skills for navigating AI tools safely. Australia and New Zealand Clinical Trials registration: ACTRN 12624000641594.

## Introduction

Generative artificial intelligence (AI) tools such as ChatGPT allow users to ask any question and immediately receive a plausible and highly tailored response. The uptake of generative AI tools has increased steadily over time, and there is now a sizable portion of the population using them. For example, an estimated 1 in 10 Australians reported using ChatGPT to ask health questions in the first half of 2024, with a further 3 in 10 considering asking it a health question in the next 6 months^1^. In this study, more than half of the people using ChatGPT for health had asked a question that would usually require clinical advice, such as diagnosis or triage. Since then, Google has added an ‘AI overview’ to search results in over 100 countries, bringing generative AI to one of the most widely used internet tools^2^. Several commentaries have expressed a hesitant optimism about the increasingly prominent role of generative AI in health communication, highlighting the potential opportunities and risks^3–5^.

On the one hand, generative AI tools have the potential to support informed health decision-making by improving the accessibility of health information. Several studies have now shown that free tools such as ChatGPT can significantly improve the readability of health information^6,7^, with newer models such as GPT-4 delivering content that meets grade reading level targets whilst maintaining a high level of accuracy^8^. This is an important capability as research consistently reports that online health information is overly complex^9^.

However, research shows that the accuracy of generative AI tools can vary depending on several factors. For example, some reviews have suggested that accuracy tends to be higher for general or factual health questions, and lower for questions that require critical thinking, situational awareness (context of personal/social situation and medical history), awareness of regional guidelines, specific treatment recommendations, and questions relating to rare diseases^6,10–14^. The accuracy can also vary according to the types of prompts and evaluation metrics used^11^. Errors and hallucinations (made-up facts) do occur and pose a genuine risk to patient safety, such as errors in medication instructions^15^. These issues are particularly pertinent for people using generic tools such as ChatGPT, as these models have not been specifically trained to answer health queries, nor do they draw on a specialised medical knowledge base.

As generative AI tools become more widely used, the public will need to acquire the knowledge and skills to help them take advantage of these tools whilst also avoiding the potential risks. It is critical that efforts to improve knowledge and skills are accessible to people with limited health literacy and who do not speak the dominant language of the country they live in, as these groups are more likely to use these tools for health tasks^1,16^ and stand to benefit substantially from access to free, tailored, understandable, and translated health content. Although frameworks and interventions for digital health literacy are increasingly common^17,18^, none of these to date have explicitly considered the skills needed to navigate generative AI tools safely^19^.

This study sought to be a starting point for bridging this research gap by co-designing and evaluating two brief health literacy interventions. These educational interventions aim to equip users with foundational knowledge about ChatGPT and the skills to reflect on the potential risks and benefits of using it to answer their health questions. We hypothesised that animation and image-based educational resources would improve AI health literacy skills relative to controls. Given that previous health literacy intervention studies have shown that visual-based, and in particular, animations or videos, are often more effective than text-based resources^20–22^, we also hypothesised that the animation would be more effective than the image-based resource.

## Results

**Fig. 1:**
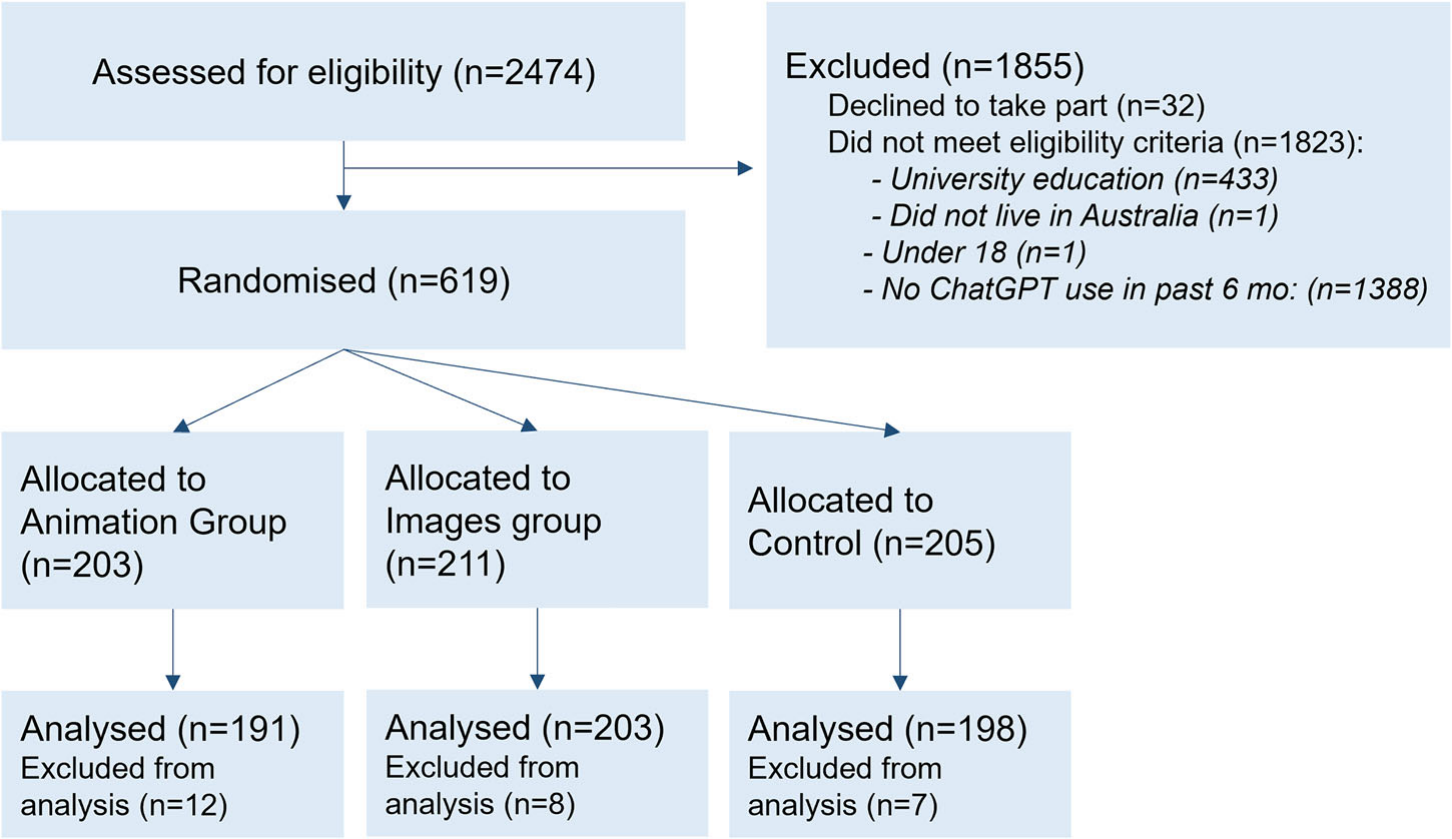
Participant flow and study design. Figure created using Microsoft PowerPoint.

Six hundred nineteen participants were randomised to an intervention or control group, and the analysis sample of 592 participants excluded 27 who did not meet fraud mitigation criteria (Fig. 1).

Descriptive statistics for the analysis sample are shown in Table 1. Two hundred and fifty-two participants (42.6%) identified as man or male, the average age was 47.0 years (SD = 16.4), and 17.4% had limited/marginal health literacy using the screening item (*n* = 103). On average, participants scored above the midpoint of the self-reported digital health literacy scale (*M* = 3.7/5, SD = 0.7). All participants had used ChatGPT in the past 6 months, and two-fifths had not asked the tool a health question during this period (*n* = 245, 41.4%). A similar proportion reported asking health questions a few times during this period (39.5%, *n* = 234). Participants reported on average ‘somewhat’ trusting ChatGPT’s advice (*M* = 3.00/5, SD = 0.91). The most common health questions that they considered asking ChatGPT related to learning about a health condition (33.8%; *n* = 200), finding out what symptoms meant (30.4%, *n* = 180), and understanding medical terms (23.6%, *n* = 140).

**Table 1:** Participant characteristics, *N* = 592

Characteristic	Animation (*n* = 191)		Images (*n* = 211)		Control (*n* = 205)	
	*n*	%	*n*	%	*n*	%
Age group (years)						
18–24	16	8.4	17	8.4	20	11.0
25–34	34	17.8	40	19.7	36	19.8
35–44	35	18.3	41	20.2	36	19.8
45–54	31	16.2	38	18.7	40	22.0
55–64	34	17.8	33	16.3	28	15.4
65+	41	21.5	34	16.7	38	20.9
Gender						
Man or male	81	42.4	90	44.3	81	44.5
Woman or female	110	57.6	112	55.2	116	63.7
Non-binary/different term	0	0.0	1	0.5	1	0.5
Education						
Less than Year 12	27	14.1	20	9.9	20	11
Year 12 or equivalent	55	28.8	82	40.4	66	36.3
Trade or technical certificate or diploma	109	57.1	101	49.8	112	61.5
Country of birth						
Australia	159	83.2	164	80.8	168	92.3
Mainly English speaking country	16	8.4	15	7.4	10	5.5
Mainly non-English speaking country	16	8.4	24	11.8	20	11.0
Language spoken at home						
English	185	96.9	187	92.1	192	105.5
Other language	6	3.1	16	7.9	6	3.3
Aboriginal and/or Torres Strait Islander						
Yes	8	4.2	3	1.5	7	3.8
No	181	94.8	199	98	190	104.4
Prefer not to say	2	1.0	1	0.5	1	0.5
Health literacy						
Limited/marginal health literacy	39	20.4	31	15.3	33	18.1
Adequate health literacy	152	79.6	172	84.7	165	90.7
Long-term health condition						
None	103	53.9	119	58.6	112	61.5
One	61	31.9	62	30.5	61	33.5
Two	23	12.0	18	8.9	15	8.2
Three or more	4	2.1	4	2.0	10	5.5
ChatGPT use in past 6 months						
A few times	123	64.4	122	60.1	147	80.8
Once a month	11	5.8	20	9.9	13	7.1
Once a week	30	15.7	25	12.3	22	12.1
More than once a week	27	14.1	36	17.7	16	8.8
ChatGPT use in past 6 months to answer questions about health						
Not at all	87	45.5	79	38.9	79	43.4
A few times	68	35.6	78	38.4	88	48.4
Once a month	17	8.9	21	10.3	16	8.8
Once a week	7	3.7	17	8.4	10	5.5
More than once a week	12	6.3	8	3.9	5	2.7

	*M*	SD	*M*	SD	*M*	SD
eHealth literacy (out of 5)	3.7	0.6	3.7	0.7	3.7	0.7
Trust in ChatGPT (baseline) (out of 5)	3.1	0.9	3.0	0.9	3.0	0.9
**Total**	**191**		**203**		**198**	

**Fig. 2:**
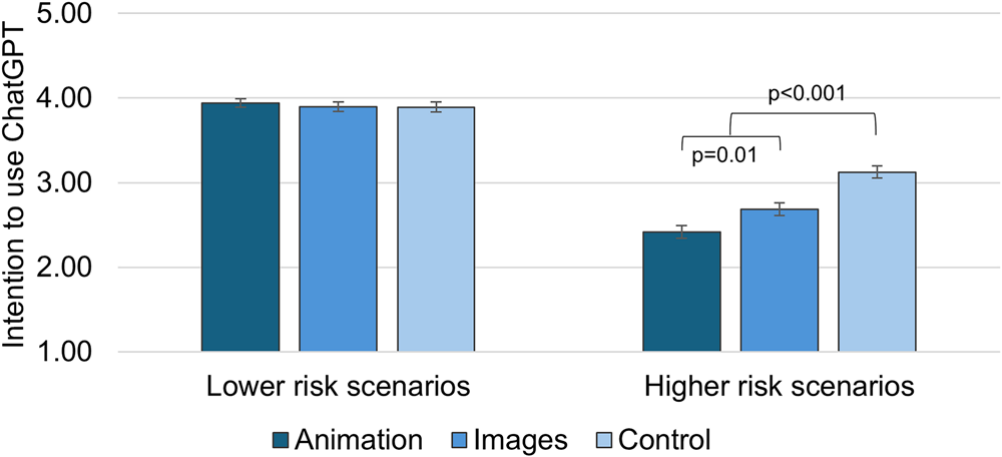
Intention to use ChatGPT, by intervention group and scenario type. Error bars indicate ±1 SE. Higher scores indicate greater intention (possible range 1–5). Figure created using Microsoft Excel.

There was no effect of group on intention to use ChatGPT for lower-risk scenarios (*F*_(2,589)_ = 0.22, *p* = 0.800; Table 2, Fig. 2). There was a significant effect of group on intention to use ChatGPT for higher-risk scenarios (*F*_(2,589)_ = 23.30, *p* < 0.001; Table 2, Fig. 2). Participants in the animation group reported lower intention to use ChatGPT for higher-risk scenarios (*M* = 2.42, SE = 0.07, 95% CI: 2.27–2.56) compared to those in the images group (*M* = 2.69, SE = 0.07, 95% CI: 2.54–2.83, *p* = 0.010; Table S1). Participants in both intervention groups reported lower intentions to use ChatGPT for higher-risk scenarios compared to the control (*M* = 3.12, SE = 0.07, 95% CI: 2.98–3.27, *p* < 0.001; Table S1).

**Table 2:** Intention to use ChatGPT for health questions by intervention group^a^

ChatGPT health scenario	Animation		Images		Control	
	*M*	SD	*M*	SD	*M*	SD
Learning about a health condition	4.23	0.89	4.11	1.08	4.06	1.06
Learning about treatment for a health condition	3.94	0.93	4.03	1.00	4.08	0.92
Asking for advice about a specific treatment	2.24	1.19	2.50	1.22	2.98	1.19
Asking about what a blood test measures	3.96	1.03	3.92	1.08	3.84	0.98
Asking to interpret a blood test result	2.62	1.23	2.80	1.28	3.26	1.18
Asking to simplify reputable information	3.63	1.06	3.53	1.12	3.60	0.99
Asking about whether to see a doctor based on symptoms	2.39	1.29	2.76	1.40	3.13	1.22
**Lower-risk scenarios**	**3.94**	**0.72**	**3.90**	**0.84**	**3.89**	**0.81**
**Higher risk scenarios**	**2.42**	**1.02**	**2.69**	**1.09**	**3.12**	**0.99**

^a^Higher scores indicate higher intention of using ChatGPT. A score of 1 refers to ‘definitely avoid’ and a score of 5 refers to ‘definitely try’ to use ChatGPT to answer the health question in the scenario(s). Scenarios categorised as higher risk are shown in grey.

**Fig. 3:**
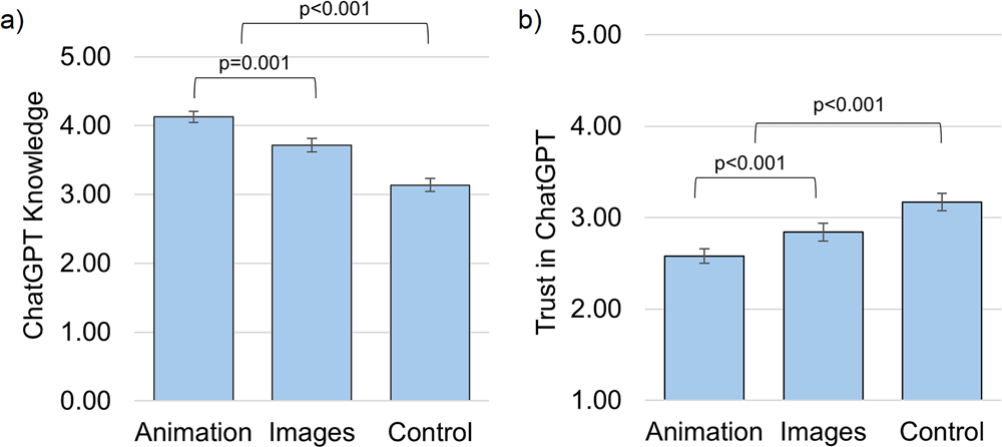
ChatGPT knowledge and trust, by intervention group. **a** Mean scores for ChatGPT knowledge; **b** means scores for trust in ChatGPT. Possible knowledge score range is 0–5. Possible trust score range is 1–5, where higher scores indicate greater trust. This figure shows estimated marginal means for trust at follow-up, controlling for trust at baseline. Figure created using Microsoft Excel.

There was a significant effect of group on ChatGPT knowledge scores (*F*_(2,589)_ = 29.422, *p* < 0.001). Participants in the animation group obtained higher ChatGPT knowledge scores (*M* = 4.13, SE = 0.09, 95% CI: 3.94–4.31) than those in the image group (*M* = 3.7, SE = 0.09, 95% CI: 3.54–3.89, *p* = 0.001; Fig. 3a, Tables S1–S3). Participants in both intervention groups obtained higher knowledge scores than those in the control group (*M* = 3.14, SE = 0.09, 95% CI: 2.96–3.31, *p* < 0.001; Tables S1–S3).

There was also a significant effect of group on trust in ChatGPT (*F*_(2,588)_ = 34.018, *p* < 0.001). Controlling for baseline trust in ChatGPT, participants in the animation group reported lower trust in ChatGPT (*M* = 2.58, SE = 0.05, 95% CI: 2.48–2.68) than those in the image group (*M* = 2.84, SE = 0.05, 95% CI: 2.74–2.94, *p* < 0.001; Fig. 3b, Tables S1 and S2). Participants in both intervention groups reported lower trust in ChatGPT than those in the control group (*M* = 3.17, SE = 0.05, 95% CI: 3.07–3.27, *p* < 0.001; Tables S1 and S2).

Across all groups, participants with limited/marginal health literacy, low self-reported digital health literacy, and low (baseline) trust in ChatGPT on average had lower intentions to use ChatGPT in lower-risk scenarios (Tables S4 and S5). We observed a significant interaction effect between group and age group (*F*_(2,586)_ = 7.11, *p* = 0.001). Younger participants (aged 18–44 years) in the control group reported lower intentions to use ChatGPT for lower-risk scenarios (*M* = 3.65, 95% CI: 3.49–3.81) compared to older participants (45 years or more) (*M* = 4.10, 95% CI: 3.95–4.25; *p* < 0.001; Tables S4 and S6; Fig. S1).

For higher-risk scenarios, we did not observe any significant main effects or interaction effects for health literacy, self-reported digital health literacy, gender, or age group (Table S4). We observed a significant interaction effect between group and baseline trust in ChatGPT (*F*_(2,586)_ = 5.00, *p* = 0.007), such that participants in the control group and images group with low trust in ChatGPT reported lower intentions to use ChatGPT for higher-risk scenarios, compared to people in those respective groups with high trust in ChatGPT (Tables S4 and S5; Fig. S2).

Participants rated the perceived effectiveness of the animation as 3.87/5 (SD = 0.67) perceived effectiveness of the images as 3.56/5 (SD = 0.74). Participants in both intervention groups rated their intentions to share the resource with friends or family above the midpoint of the scale (Animation: *M* = 4.77/7, SD = 1.4; Images: *M* = 4.45, SD = 1.51). Participants rated both the animation and the images as useful (Animation: *M* = 4.07/5, SD = 0.61; Images: *M* = 3.93/5, SD = 0.62).

## Discussion

This study provides world-first preliminary evidence that co-designed health literacy interventions can help people develop skills to critically appraise which kinds of health questions are riskier to ask ChatGPT. In line with the intervention content, people allocated to the animation and images intervention groups had lower intentions to ask ChatGPT health questions that are riskier, such as asking for personalised treatment advice, interpreting test results, or symptom diagnosis, relative to people in the control group. Their intentions to ask lower-risk health questions were similar to those of people in the control group. People allocated to the intervention groups also demonstrated significantly higher knowledge of ChatGPT and its limitations, and lower trust in the tool. These effects were more pronounced for people who viewed the animation compared to the images.

Reliance on ChatGPT and other generative AI tools as a source of health information is a contentious issue^3,4^. Whilst there are clear benefits, such as their ability to simplify health information^8^, this must be balanced against the potential safety risks^11^. The current study demonstrates that brief online interventions can develop AI health literacy skills and improve community use of this new technology. However, the exact nature of AI health literacy skills is likely to change as the technology advances. For example, ChatGPT can now integrate real information sources into its answers^23^. This feature overcomes some of the tool’s key limitations, though the quality of the information sources remains a concern and still requires users to have critical appraisal skills^24^.

This study has several strengths and limitations. As described above, the project was initiated by a consumer and co-designed. We recruited a sample of participants who did not have an undergraduate level of education. This group is more likely to have limited/marginal health literacy and stand to benefit from interventions that incorporate health literacy principles^25^.

The study also has several limitations. There were no existing appropriate validated performance-based assessments of AI health literacy or ChatGPT knowledge that were available at the time of the study, though there is a validated assessment of self-reported ChatGPT literacy that may be useful in evaluations of these kinds of resources^26^. Although we developed our own purpose-designed instrument, its psychometric properties have not been evaluated; and changes in intentions may not necessarily translate to changes in behaviour, a well-documented phenomenon^27^. We also acknowledge that the interventions were tested in a controlled setting, requiring participants to be exposed to the intervention for a specified time period. It is not clear how effective these interventions would be in real-life settings. For example, if implemented directly through social media, they may not compete effectively with the vast amount of ChatGPT content that rapidly adapts to incorporate trends in user-generated content. Strategies such as shorter videos, humour or memes, and content creation by influencers may increase engagement and intention to share the content^28^, or alternatively, the videos could be delivered in settings where longer content is more appropriate e.g. primary care clinics.

Future work could consider enhancing the content in several ways. The content could be updated for changes to ChatGPT technology and a broader skillset e.g. strategies to support preparation for medical consultations, adapted for other AI tools such as Gemini or Co-Pilot; and focus on cultivating broader skills to help people decide which AI tools to use. The content could also be adapted for people in culturally and linguistically diverse communities and translated into other languages, as we know from previous research that these groups are more likely to ask ChatGPT health questions^1^. Further, as a complex intervention, it is not clear which specific elements contributed to the effects, and we only tested two versions of the content. Future work could consider head-to-head testing of a variety of different formats e.g. text only, video content, and variations within each format e.g. shorter and longer videos, voice speed, to optimise these resources.

Our findings demonstrate that brief health literacy interventions may help improve knowledge of ChatGPT and reduce intentions to ask riskier health questions. The interventions specifically targeted ChatGPT use; as such, this study represents an initial step towards addressing AI health literacy in the community. It highlights the kinds of health literacy skills that can support people to navigate AI tools more safely. With the rapid uptake of AI tools such as ChatGPT in the community, it is crucial that health literacy and public health research keeps pace. This means developing equitable and inclusive resources to equip the community with the skills they need to suit the changing digital environment.

## Methods

### Study design

This was a co-designed three-arm parallel-group randomised controlled trial study design with participants randomly allocated to a group in the ratio 1:1:1 (Fig. 1). This trial was prospectively registered with the Australian New Zealand Clinical Trials Registry (ACTRN 12624000641594) on 27 May 2024 and approved by the University of Sydney Human Research Ethics Committee (2024/HE000247). CONSORT and TIDieR checklists are available in Supplementary Information A. This online study was available through the Qualtrics platform.

### Intervention development

Increasingly, there is a strong imperative to engage with consumers (public, patients, and carers) in the development of health resources. Key bodies, including the National Institute for Health and Care Research in the UK^29^, and the Patient-Centred Outcomes Research Institute in the US^30^ (funded by the Patient Protection and Affordable Care Act 2010), highlight consumer engagement as a key component of high-quality research practice, and there are also widely established strong ethical arguments to support this approach^31^. As such, in this study, we used a particular form of consumer engagement, ‘co-design,’ to develop the study materials in partnership with community member research partners, herein referred to as ‘community members.’ Our process is outlined below.

The research question was initially proposed by a community member after reflecting on a previous ChatGPT study that they had contributed to^7^. We then partnered with six Australian community members with diverse experiences (age, gender, cultural background, metropolitan vs rural, and familiarity with ChatGPT) to develop the study design and materials. The community members became involved through their role in the research lab’s community panel, which supports consumer engagement activities within the broader health literacy research programme. As the resources were intended for the general Australian public, there was no specific lived experience required.

Members met online across 4 meetings between November 2023 and May 2024 (Table S7). Community involvement was integral to refining key messages (Table S2), co-development of resources, and designing the study, including purpose-built outcome measures to evaluate knowledge and skills. A fifth meeting sought to integrate community perspectives into the interpretation of the results.

When designing the resources, we also incorporated health literacy guidelines wherever possible e.g. using informative headings (or signposting for audiovisual mediums), using common, everyday language, aiming for a Grade 8 reading level, and using the active voice^32,33^. The initial images and script/storyboard for the video were drafted in Canva and Microsoft Word/PowerPoint, respectively, to facilitate discussion with community members. After finalising the script and storyboard for the animation, the content for both resources was then given to a professional designer. The research team, including the community members, provided ongoing feedback to the designer to support iterative revisions of the resources.

### Participants

Eligible participants were adults (18+ years) living in Australia who did not have a university-level education and who reported using ChatGPT in the past 6 months. Participants were recruited between 20 November and 16 December 2024, through PureProfile, which manages a member panel of over 550,000 Australian adults. People in the community can decide to sign up for the panel and take part in studies in return for points that can be redeemed for cash, gift cards, and other benefits. Survey invitations are standardised and give no indication of the survey content.

This study focuses on people without a university education, as the resources are intended to provide accessible, easy-to-understand information suitable for people with low literacy. Whilst we acknowledge that education is not a direct proxy for literacy, the two concepts are interrelated^25^, and this eligibility criterion was used to identify potential participants who may benefit more greatly from the educational content.

### Groups

The key messages that were conveyed in the intervention resources were developed between November 2023 to May 2024, and reflect the capabilities of ChatGPT during this period (Table S8). These key messages continued to be of relevance during the recruitment period. Both interventions (images and animation) covered the same content. The interventions can be viewed at Open Science Framework^34^.

### Animation intervention group

Participants viewed an animation (3 min 40 s) that included a voiceover and closed captions. The script was written at a Grade 9 reading level and contained 7.3% complex language and 1 passive voice, as assessed by the Sydney Health Literacy Lab Health Literacy Editor^33^. Participants had the ability to pause, replay, and change video speed.

### Image intervention group

Participants viewed a series of 7 images embedded within an image carousel, similar to what appears on the social media platform Instagram. In addition to the key messages shown in Table S8, the images showed examples of questions that you might try or avoid asking ChatGPT. The text was written at a Grade 7.8 reading level and contained 7.3% complex language, with no passive voice. Participants were able to move backwards and forwards through the images.

### Control

Participants viewed an infographic on healthy eating that had been developed by the Australian Government^35^. This infographic was selected as a control as it was a neutral health stimulus that did not contain any content related to ChatGPT or online health information seeking.

### Procedure

Piloting took place between 5 July and 7 November 2024, and ensured that recruitment methods were feasible and refined the phrasing of the primary outcome items to ensure they were clear.

Potential participants were invited to take part via the PureProfile portal, with links to the survey hosted on Qualtrics. After consenting, participants completed demographic items, a health literacy screening item^36^ and a measure of self-reported digital health literacy (eHeals)^37^. Participants were asked how much they trusted ChatGPT, how often they had used ChatGPT in the past 6 months, and what kinds of health questions they had asked ChatGPT (Supplementary Information C).

Participants were then randomised to a group and asked to view the materials. Participants in the animation group were required to remain on the page for the length of the video (3 min 40 s). Participants in the image and control groups were required to remain on the page for 60 s. All participants then completed primary and secondary outcomes, and those in the intervention groups completed questions about the acceptability of the intervention. Participants who completed the survey received reimbursement for their participation.

### Outcomes

The primary outcomes were intentions to use ChatGPT for lower and higher-risk health questions. These were based on responses to 7 purpose-built, scenario-based items that asked participants to indicate their intentions to ask different health questions to ChatGPT (5-point Likert scale: definitely avoid to definitely try). Scenarios for each item are shown in Appendix C. Items 1, 2, 4 and 6 were considered lower risk as they focused on scenarios about general health knowledge e.g. ‘*What is gout?’*. Items 3, 5 and 7 were considered higher risk as they focused on scenarios asking for personalised advice about treatment, pathology and diagnosis, that would usually require clinical interpretation e.g. ‘*Should my uncle use corticosteroids to treat gout?’*. These scores assessed intention to use ChatGPT for higher-risk scenarios. Though a measure of intention, scores for this outcome reflect participants’ ability to apply their ChatGPT knowledge and skills to novel hypothetical health scenarios. Cronbach’s α was 0.784 for lower-risk scenarios and 0.792 for higher-risk scenarios.

There were two secondary outcomes; participants also completed a 6-item assessment of ChatGPT knowledge (Supplementary Information C). These items mirrored key messages in the intervention content and were marked as correct or incorrect. Review of items prior to analysis identified that the wording of one item was unclear. The inclusion of this item also impacted the reliability of the measure (Cronbach’s *α* = 0.542); for these two reasons, this item was excluded from this analysis. The remaining 5 items were included. Cronbach’s α was 0.701. Participants repeated the baseline assessment of trust in ChatGPT after viewing the interventions (5-point Likert scale).

Those allocated to the two intervention groups completed items relating to intervention acceptability (Supplementary Information C). Perceived effectiveness was assessed via 6 descriptors (worth-remembering, attention-grabbing, powerful, informative, meaningful and convincing), with responses recorded on a 5-point Likert scale^38^. Intention to share the intervention on social media was collected using a single item adapted from another digital health literacy intervention study (7-point Likert scale)^39^. Seven items assessed perceived usefulness of the intervention content, with responses rated using a 5-point Likert scale. Items asked about the novelty of the intervention, its impact, future intentions to act on advice in the intervention, and confidence in the intervention content. This was adapted from the same digital health literacy intervention study^39^. All scales ranged from strongly disagree to strongly agree.

### Analysis

Planned orthogonal contrasts between the two intervention arms and the control arm were implemented in regression models (i.e. interventions vs control; animation vs images). We applied the Bonferroni correction to significance testing thresholds for the two primary outcomes and two secondary outcomes (*p* < 0.0125). For the secondary outcome ‘trust in ChatGPT,’ the baseline assessment of the same item was included as a covariate in the model. Exploratory analyses examined the influence of health literacy, digital literacy, gender, age, and trust in ChatGPT by including interaction terms within the regression models. These were analysed as binary categories. Categories for health literacy reflect the cut-offs suggested by Wallace et al.^36^ (i.e. for limited/marginal and adequate). Scores below the midpoint of the eHeals scale were considered ‘low’ digital health literacy. For trust in ChatGPT, scores of ‘somewhat’ or below were considered ‘low.’ Two age categories (18–44 and 45+) were used as these age groups observed varied use of ChatGPT for health in a previous study^1^. Significant interactions were explored further using simple post-hoc comparisons. Outcomes related to acceptability were analysed descriptively.

In line with existing fraud mitigation strategies, participants were excluded from analyses if they completed survey questions in a short timeframe (i.e. less than 2 min for survey questions estimated to take 9 min) or failed to meet built-in Qualtrics quality checks e.g. ReCAPTCHA scores^40^. All participants who met eligibility and fraud mitigation criteria passed the commitment check.

### Sample size

The total sample size required was 567. This was based on an expected small effect size of 0.15 for the primary outcome. The number of participants required per arm (3 arms), to provide 90% power at error type I (alpha = 0.05) was 189 (this is a balanced design).

## Supplementary information

Supplementary Information

## Data Availability

The datasets analysed during the current study are available from the corresponding author on reasonable request
