# Supplementary material for: Can co-designed educational interventions help consumers think critically about asking ChatGPT health questions? Results from a randomised-controlled trial
Source: NPJ Digit Med. 2025 Nov 17;8:672. doi: 10.1038/s41746-025-02056-5 (PMC12623733; doi:10.1038/s41746-025-02056-5)
Supplement: Supplementary file 1 — Supplementary Information [file 41746_2025_2056_MOESM1_ESM.pdf]

Can a co-designed educational intervention help consumers think critically about asking ChatGPT health questions? Results from a randomised-controlled trial

Supplementary information A – CONSORT and TIDieR checklists

Section/topic	No	CONSORT 2025 checklist item description	Reported on page no.
**Title and abstract**			
Title and structured abstract	1a	Identification as a randomised trial	1
	1b	Structured summary of the trial design, methods, results, and conclusions	2
**Open science**			
Trial registration	2	Name of trial registry, identifying number (with URL) and date of registration	11
Protocol and statistical analysis plan	3	Where the trial protocol and statistical analysis plan can be accessed	NA
Data sharing	4	Where and how the individual de-identified participant data (including data dictionary), statistical code and any other materials can be accessed	17
Funding and conflicts of interest	5a	Sources of funding and other support (eg, supply of drugs), and role of funders in the design, conduct, analysis and reporting of the trial	18
	5b	Financial and other conflicts of interest of the manuscript authors	18
**Introduction**			
Background and rationale	6	Scientific background and rationale	3-4
Objectives	7	Specific objectives related to benefits and harms	4-5
**Methods**			
Patient and public involvement	8	Details of patient or public involvement in the design, conduct and reporting of the trial	11-12
Trial design	9	Description of trial design including type of trial (eg, parallel group, crossover), allocation ratio, and framework (eg, superiority, equivalence, non-inferiority, exploratory)	10-11
Changes to trial protocol	10	Important changes to the trial after it commenced including any outcomes or analyses that were not prespecified, with reason	Supp Inf B, 15-16
Trial setting	11	Settings (eg, community, hospital) and locations (eg, countries, sites) where the trial was conducted	12-13
Eligibility criteria	12a	Eligibility criteria for participants	12-13
	12b	If applicable, eligibility criteria for sites and for individuals delivering the interventions (eg, surgeons, physiotherapists)	NA
Intervention and comparator	13	Intervention and comparator with sufficient details to allow replication. If relevant, where additional materials describing the intervention and comparator (eg, intervention manual) can be accessed	13-14

			Reported on page no.
Outcomes	14	Prespecified primary and secondary outcomes, including the specific measurement variable (eg, systolic blood pressure), analysis metric (eg, change from baseline, final value, time to event), method of aggregation (eg, median, proportion), and time point for each outcome	15-16
Harms	15	How harms were defined and assessed (eg, systematically, non-systematically)	NA
Sample size	16a	How sample size was determined, including all assumptions supporting the sample size calculation	17
	16b	Explanation of any interim analyses and stopping guidelines	NA
Randomisation:			
Sequence generation	17a	Who generated the random allocation sequence and the method used	10, 14
	17b	Type of randomisation and details of any restriction (eg, stratification, blocking and block size)	10, 14
Allocation concealment mechanism	18	Mechanism used to implement the random allocation sequence (eg, central computer/telephone; sequentially numbered, opaque, sealed containers), describing any steps to conceal the sequence until interventions were assigned	14 (Qualtrics randomizer)
Implementation	19	Whether the personnel who enrolled and those who assigned participants to the interventions had access to the random allocation sequence	NA (automated)
Blinding	20a	Who was blinded after assignment to interventions (eg, participants, care providers, outcome assessors, data analysts)	14
	20b	If blinded, how blinding was achieved and description of the similarity of interventions	NA
Statistical methods	21a	Statistical methods used to compare groups for primary and secondary outcomes, including harms	16-17
	21b	Definition of who is included in each analysis (eg, all randomised participants), and in which group	Figure 1
	21c	How missing data were handled in the analysis	No missing data
	21d	Methods for any additional analyses (eg, subgroup and sensitivity analyses), distinguishing prespecified from post hoc	16-17
**Results**			
Participant flow, including flow diagram	22a	For each group, the numbers of participants who were randomly assigned, received intended intervention, and were analysed for the primary outcome	Figure 1
	22b	For each group, losses and exclusions after randomisation, together with reasons	Figure 1
Recruitment	23a	Dates defining the periods of recruitment and follow-up for outcomes of benefits and harms	12
	23b	If relevant, why the trial ended or was stopped	NA
Intervention and comparator delivery	24a	Intervention and comparator as they were actually administered (eg, where appropriate, who delivered the intervention/comparator, how participants adhered, whether they were delivered as intended (fidelity))	Figure 1
	24b	Concomitant care received during the trial for each group	NA
Baseline data	25	A table showing baseline demographic and clinical characteristics for each group	Table 1
Numbers analysed, outcomes and estimation	26	For each primary and secondary outcome, by group:<br>● the number of participants included in the analysis<br>● the number of participants with available data at the outcome time point<br>● result for each group, and the estimated effect size and its precision (such as 95% confidence interval)<br>● for binary outcomes, presentation of both absolute and relative effect size	Figure 1, 14 Supp Info Tables
Harms	27	All harms or unintended events in each group	NA

Ancillary analyses	28	Any other analyses performed, including subgroup and sensitivity analyses, distinguishing pre-specified from post hoc	7, Appendix Tables
**Discussion**			
Interpretation	29	Interpretation consistent with results, balancing benefits and harms, and considering other relevant evidence	8-10
Limitations	30	Trial limitations, addressing sources of potential bias, imprecision, generalisability, and, if relevant, multiplicity of analyses	9

Citation: Hopewell S, Chan AW, Collins GS, Hróbjartsson A, Moher D, Schulz KF, et al. CONSORT 2025 Statement: updated guideline for reporting randomised trials. BMJ. 2025; 388:e081123. https://dx.doi.org/10.1136/bmj-2024-081123
© 2025 Hopewell et al. This is an Open Access article distributed under the terms of the Creative Commons Attribution License (https://creativecommons.org/licenses/by/4.0/), which permits unrestricted use, distribution, and reproduction in any medium, provided the original work is properly cited.

*We strongly recommend reading this statement in conjunction with the CONSORT 2025 Explanation and Elaboration and/or the CONSORT 2025 Expanded Checklist for important clarifications on all the items. We also recommend reading relevant CONSORT extensions. See www.consort-spirit.org.

The TIDieR (Template for Intervention Description and Replication) Checklist*:

Information to include when describing an intervention and the location of the information

Item number	Item	Where located ** Primary paper (page or appendix number)	Other † (details)
	**BRIEF NAME**		
**1.**	Provide the name or a phrase that describes the intervention.	______page 13	______________
	**WHY**		
**2.**	Describe any rationale, theory, or goal of the elements essential to the intervention.	______page 11-13	______________
	**WHAT**		
**3.**	Materials: Describe any physical or informational materials used in the intervention, including those provided to participants or used in intervention delivery or in training of intervention providers. Provide information on where the materials can be accessed (e.g. online appendix, URL).	______page 11-13	______________
**4.**	Procedures: Describe each of the procedures, activities, and/or processes used in the intervention, including any enabling or support activities.	_______None	______________
	**WHO PROVIDED**		
**5.**	For each category of intervention provider (e.g. psychologist, nursing assistant), describe their expertise, background and any specific training given.	Online (page 14-15)	______________
	**HOW**		
**6.**	Describe the modes of delivery (e.g. face-to-face or by some other mechanism, such as internet or telephone) of the intervention and whether it was provided individually or in a group.	Online, individually (page 14)	______________
	**WHERE**		
**7.**	Describe the type(s) of location(s) where the intervention occurred, including any necessary infrastructure or relevant features.	Online survey (page 14)	______________

	**WHEN and HOW MUCH**		
**8.**	Describe the number of times the intervention was delivered and over what period of time including the number of sessions, their schedule, and their duration, intensity or dose.	Page 14	______________
	**TAILORING**		
**9.**	If the intervention was planned to be personalised, titrated or adapted, then describe what, why, when, and how.	N/A	______________
	**MODIFICATIONS**		
**10.**ǂ	If the intervention was modified during the course of the study, describe the changes (what, why, when, and how).	None	______________
	**HOW WELL**		
**11.**	Planned: If intervention adherence or fidelity was assessed, describe how and by whom, and if any strategies were used to maintain or improve fidelity, describe them.	None	______________
**12.**ǂ	Actual: If intervention adherence or fidelity was assessed, describe the extent to which the intervention was delivered as planned.	None – minimum time on page was required (see page 14)	______________

** **Authors -** use N/A if an item is not applicable for the intervention being described. **Reviewers** – use '?' if information about the element is not reported/not sufficiently reported.

† If the information is not provided in the primary paper, give details of where this information is available. This may include locations such as a published protocol or other published papers (provide citation details) or a website (provide the URL).

ǂ If completing the TIDieR checklist for a protocol, these items are not relevant to the protocol and cannot be described until the study is complete.

* We strongly recommend using this checklist in conjunction with the TIDieR guide (see *BMJ* 2014;348:g1687) which contains an explanation and elaboration for each item.

* The focus of TIDieR is on reporting details of the intervention elements (and where relevant, comparison elements) of a study. Other elements and methodological features of studies are covered by other reporting statements and checklists and have not been duplicated as part of the TIDieR checklist. When a **randomised trial** is being reported, the TIDieR checklist should be used in conjunction with the CONSORT statement (see www.consort-statement.org) as an extension of **Item 5 of the CONSORT 2010 Statement.** When a **clinical trial protocol** is being reported, the TIDieR checklist should be used in conjunction with the SPIRIT statement as an extension of **Item 11 of the SPIRIT 2013 Statement** (see www.spirit-statement.org). For alternate study designs, TIDieR can be used in conjunction with the appropriate checklist for that study design (see www.equator-network.org).

Supplementary information B: Supplementary tables and figures

Table S1. Orthogonal contrasts for effect of group on primary and second outcomes.

Outcome	t	df	p	Mean difference	SE (difference)	95% CI (difference)
**Intention to use ChatGPT for health questions**						
**Lower risk scenarios**						
Animation vs image	-0.563	588	0.577	-0.045	0.080	-0.201 to 0.112
Interventions vs control	-0.377	588	0.705	-0.026	0.069	-0.162 to 0.109
**Higher risk scenarios**						
Animation vs image	2.606	588	0.010	0.271	0.104	0.066 to 0.476
Interventions vs control	6.378	588	<0.001	0.574	0.090	0.397 to 0.751
**Knowledge**						
Animation vs image	-3.186	588	0.001	-0.411	0.129	-0.665 to -0.158
Interventions vs control	-7.063	588	<0.001	-0.784	0.111	-1.002 to -0.565
**Trust***						
Animation vs image	3.676	588	<0.001	0.261	0.071	0.401 to 0.457
Interventions vs control	7.492	588	<0.001	0.457	0.061	0.336 to 0.577

*Analysis controlled for baseline trust (covariate).

Table S2. ChatGPT knowledge score and trust in ChatGPT at followup, by group

	Knowledge score		Trust in ChatGPT (follow-up)	
**Group**	**M**	**SD**	**M**	**SD**
Animation	4.13	1.08	2.62	0.90
Images	3.71	1.39	2.85	0.95
Control	3.14	1.34	3.13	0.93

Note: Possible knowledge score range 0 to 5. Possible trust score range 1 to 5.

Table S3. ChatGPT knowledge score by group and individual knowledge item, number and per cent correct

	Animation		Images		Control		Total	
**Knowledge item**	**n**	**%**	**n**	**%**	**n**	**%**	**n**	**%**
It's safe to use ChatGPT in an emergency if you really need to	172	90.1	155	76.4	113	57.1	440	74.3
ChatGPT can be useful for answering general health questions	177	92.7	180	88.7	171	86.4	528	89.2
If ChatGPT is confident when it tells you how to interpret your blood test results, its answer is more likely to be correct	149	78.0	138	68.0	107	54.0	394	66.6
You can trust ChatGPT's answer if it references journal articles that report on clinical trials	118	61.8	116	57.1	81	40.9	315	53.2
ChatGPT knows which treatment option is best for you	172	90.1	165	81.3	149	75.3	486	82.1

Table S4. ANOVA regression models for effect of group and selected demographic variables (health literacy, digital health literacy, baseline trust in ChatGPT, gender, and age) on intentions to use ChatGPT for health questions.

ChatGPT health scenario	Main effect: Group		Main effect: demographic variable		Interaction effect: group X demographic variable	
	F	p	F	p	F	p
**Lower risk scenarios**						
Health literacy	0.12	0.884	20.92	**<0.001**	0.16	0.853
Digital health literacy	0.47	0.628	14.62	**<0.001**	1.61	0.200
Trust in ChatGPT (baseline)	0.05	0.955	6.35	**0.012**	2.28	0.103
Age group	0.43	0.653	3.15	0.076	7.11	**0.001**
Gender	0.22	0.799	1.53	0.216	0.55	0.580
**Higher risk scenarios**						
Health literacy	11.41	**<0.001**	2.62	0.106	0.64	0.528
Digital health literacy	10.00	**<0.001**	1.53	0.217	0.29	0.752
Trust in ChatGPT (baseline)	29.45	**<0.001**	27.44	**<0.001**	5.00	**0.007**
Age group	22.03	**<0.001**	0.38	0.539	2.33	0.098
Gender	22.56	**<0.001**	0.80	0.782	1.45	0.237

Health literacy was assessed using the single item screener.[11] Digital health literacy was assessed using the eHeals.[12] The cut-off for low was based on scores below the midpoint of the scale. The effect of age group was based on participants aged 18 to 44 years vs 45+ years. The effect of gender compared man or male and woman or female. Scores indicating trusting ChatGPT 'Extremely' or 'Quite a bit' were considered 'high.' All other response options ('Not at all,' 'A little bit' and 'Somewhat') were considered low.

Table S5. Intention to use ChatGPT for health questions by group, health literacy, digital health literacy, and baseline trust in ChatGPT*

ChatGPT health scenario	Animation				Images				Control			
	M	SD	t	p	M	SD	t	p	M	SD	t	p
**Lower risk scenarios**												
Limited/marginal health literacy	3.59	0.78	-3.156	**0.002**	3.62	0.85	-2.135	**0.033**	3.56	0.92	-2.675	**0.008**
Adequate health literacy	4.03	0.68			3.95	0.83			3.96	0.77		
Low digital health literacy	3.46	0.69	-3.298	**0.001**	3.78	0.89	-0.897	0.370	3.61	0.99	-2.379	**0.018**
High digital health literacy	4.01	0.70			3.92	0.83			3.95	0.76		
Low trust in ChatGPT	3.95	0.69	0.256	0.798	3.83	0.84	-1.991	**0.047**	3.80	0.81	-2.677	**0.008**
High trust in ChatGPT	3.92	0.79			4.07	0.82			4.13	0.77		
**Higher risk scenarios**												
Limited/marginal health literacy	2.59	0.86	1.180	0.239	2.96	0.92	1.582	0.114	3.13	1.02	0.041	0.967
Adequate health literacy	2.37	1.05			2.64	1.11			3.12	0.99		
Low digital health literacy	2.39	0.79	-0.149	0.882	2.55	1.14	-0.756	0.450	2.91	0.96	-1.323	0.186
High digital health literacy	2.42	1.05			2.71	1.09			3.17	1.00		
Low trust in ChatGPT	2.36	0.97	-1.189	0.235	2.58	1.03	-2.365	**0.018**	2.87	0.95	-5.549	**<0.001**
High trust in ChatGPT	2.55	1.13			2.94	1.20			3.74	0.82		

*Higher scores indicate higher intention of using ChatGPT. A score of 1 refers to definitely avoid and a score of 5 refers to definitely try to use ChatGPT to answer the health question in the scenario(s). Health literacy was assessed using the single item screener.[11] Digital health literacy was assessed using the eHeals.[12] The cut-off for low was based on scores below the midpoint of the scale. Scores indicating trusting ChatGPT 'Extremely' or 'Quite a bit' were considered 'high.' All other response options ('Not at all,' 'A little bit' and 'Somewhat') were considered low.

Table S6. Intention to use ChatGPT for health questions by group, gender, and age group

ChatGPT health scenario	Animation				Images				Control			
	M	SD	t	p	M	SD	t	p	M	SD	t	p
**Lower risk scenarios**												
Age group: 18 to 44 years	4.01	0.71	1.117	0.264	3.89	0.78	-0.200	0.842	3.65	0.90	-4.025	**<0.001**
Age group: 45 years or more	3.88	0.73			3.91	0.89			4.10	0.67		
Gender: Man or male	3.87	0.66	-1.011	0.116	3.90	0.78	0.123	0.112	3.81	0.86	-1.234	0.218
Gender: Woman or Female	3.99	0.76			3.89	0.88			3.95	0.78		
**Higher risk scenarios**												
Age group: 18 to 44 years	2.53	1.07	1.365	0.173	2.62	1.06	-0.942	0.347	3.00	0.98	-1.534	0.126
Age group: 45 years or more	2.32	0.97			2.75	1.12			3.23	1.00		
Gender: Man or male	2.39	0.95	-0.327	0.744	2.82	1.03	1.546	0.123	3.07	0.98	-0.703	0.482
Gender: Woman or Female	2.44	1.07			2.59	1.13			3.18	1.00		

*Higher scores indicate higher intention of using ChatGPT. A score of 1 refers to definitely avoid and a score of 5 refers to definitely try to use ChatGPT to answer the health question in the scenario(s). Analysis for gender does not include participants who responded ‘Non-binary’ or other term.

Table S7. Summary of co-design activities

Meeting	Activities / aims
**1: Establish project scope**	The researchers presented an overview of ChatGPT and findings from their previous ChatGPT study.[1] The panel then completed a brainstorming activity to gather examples of using ChatGPT for health questions and confidence asking these questions. This was followed by a discussion about comparing output from Google snippets and ChatGPT. Members had opportunity to raise further questions about using ChatGPT for health questions. Finally, the panel members and researchers collaboratively described the project scope including intended audience and format of trial resources.
**2: Refine key educational messages**	Key educational messages were drafted and presented to the panel. Key topics are shown in Table 2. The panel gave feedback on the key messages and associated content, including the tone/language, caveats they felt were important, and any content that was unclear
**3: Present content and gather feedback**	Key messages were converted into a set of social media images and a script for an animation. Panel members gave feedback on these.
**4: Revise content**	Panel members viewed revised images and a draft animation, as well as feedback on survey design and drafted outcome measures. Feedback on outcome measures sought to make sure that items were relevant and meaningfully tested the content of the interventions. Panel members discussed ways to recruit participants (social media). On piloting, further changes were discussed to refine the outcome measures and identify a more feasible recruitment pathway.
**5: Present and discuss findings**	The researchers presented preliminary findings to the panel and elicited a discussion about the findings. Panel members had opportunity to view the draft paper and provide feedback. Panel members discussed potential ways to disseminate the results. Panel members were also asked to reflect on the study processes and how they were engaged as consumers.

Table S8. Key messages

Component	Key messages
**Basic ChatGPT concepts** [2]	• ChatGPT doesn't evaluate or synthesise evidence.<br>• It can be hard to know where ChatGPT gets its information from. Even when it gives sources, these can be fake or incorrect.<br>• It is risky to use ChatGPT as a single source of truth.<br>• ChatGPT's confidence is not related to the accuracy of its output.<br>• Don't use ChatGPT in an emergency
**Understanding potential risks of using ChatGPT for health** [3-8]	• General health questions are less risky e.g. understanding a medical term, a health condition, treatment options, or summarising health information from a reliable source.<br>• Answers to general questions may be incorrect, missing key points, or lack important personal context.<br>• Specific health questions that relate to personal health decisions/actions are more risky e.g. advice on treatment, diagnosis, and interpretation of medical tests.<br>• In addition to the issues for general health questions, specific questions may cause harm including unnecessary stress or worry
**Strategies to reduce the risk associated with using ChatGPT for health** [3-10]	• If you want to use ChatGPT, focus on general questions and avoid asking about which treatment to try, diagnosis, or interpretation of medical tests.<br>• Speaking with a health professional is always the safest option.<br>• Compare ChatGPT's advice to information from reliable sources.<br>• Advice for prompts: (1) Provide context where possible and that you are comfortable to share e.g. gender/age/country but not personal information; (2) Understand the data that ChatGPT uses i.e. it may be old; (3) Break down prompts into steps; (4) Experiment with different prompts including role-playing

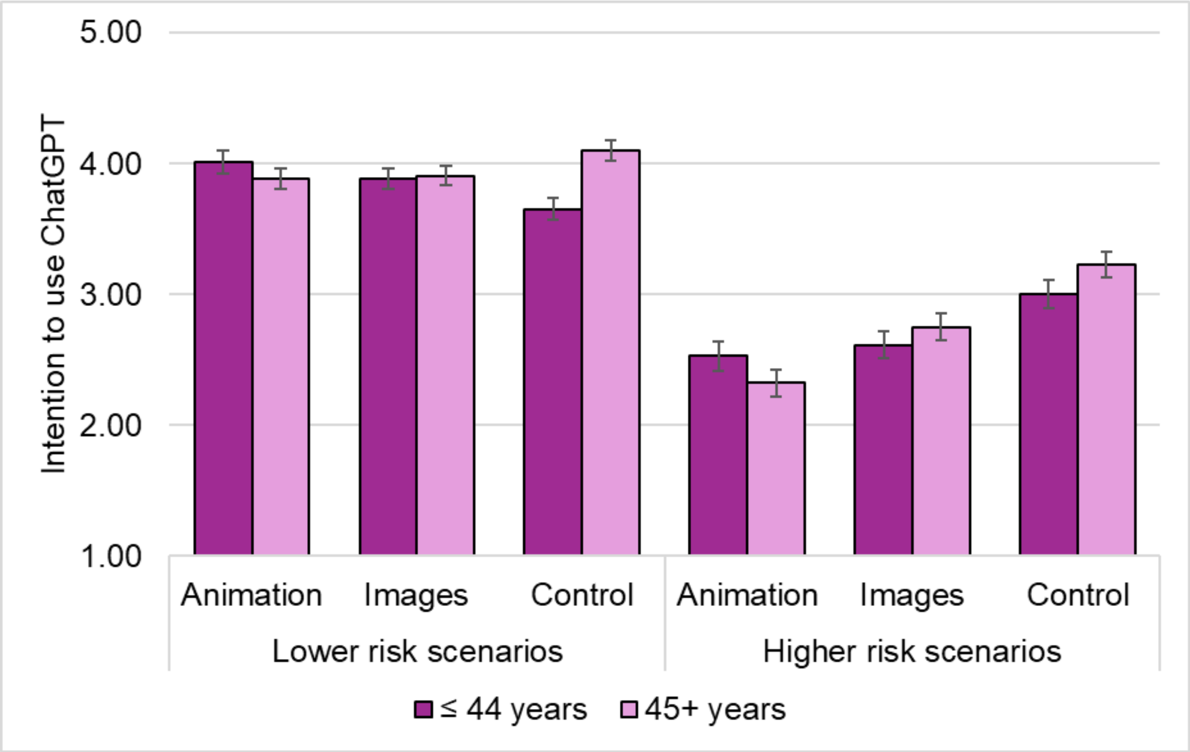

Figure S1. Intention to use ChatGPT, by group, age group and scenario type.
Note: Error bars indicate ±1 SE. Higher scores indicate higher intention of using ChatGPT. Figure created using Microsoft Excel.

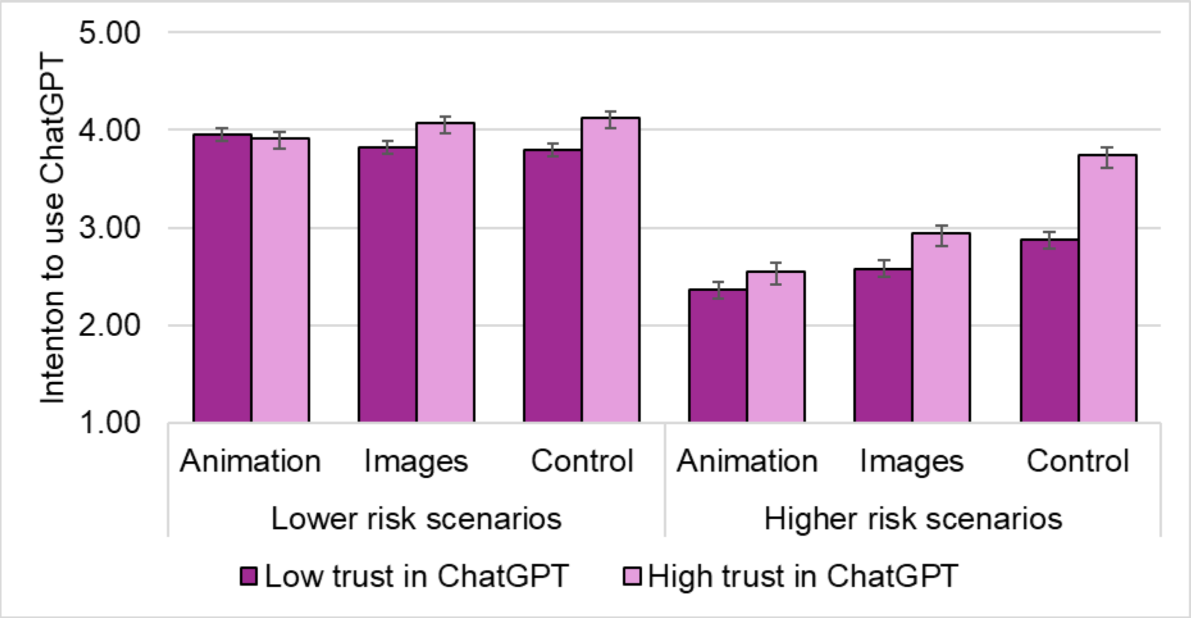

Figure S2. Intention to use ChatGPT, by group, baseline trust in ChatGPT and scenario type.
Note: Error bars indicate ±1 SE. Higher scores indicate higher intention of using ChatGPT. Scores indicating trusting ChatGPT 'Extremely' or 'Quite a bit' were considered high. All other response options ('Not at all,' 'A little bit' and 'Somewhat') were considered low. Figure created using Microsoft Excel.

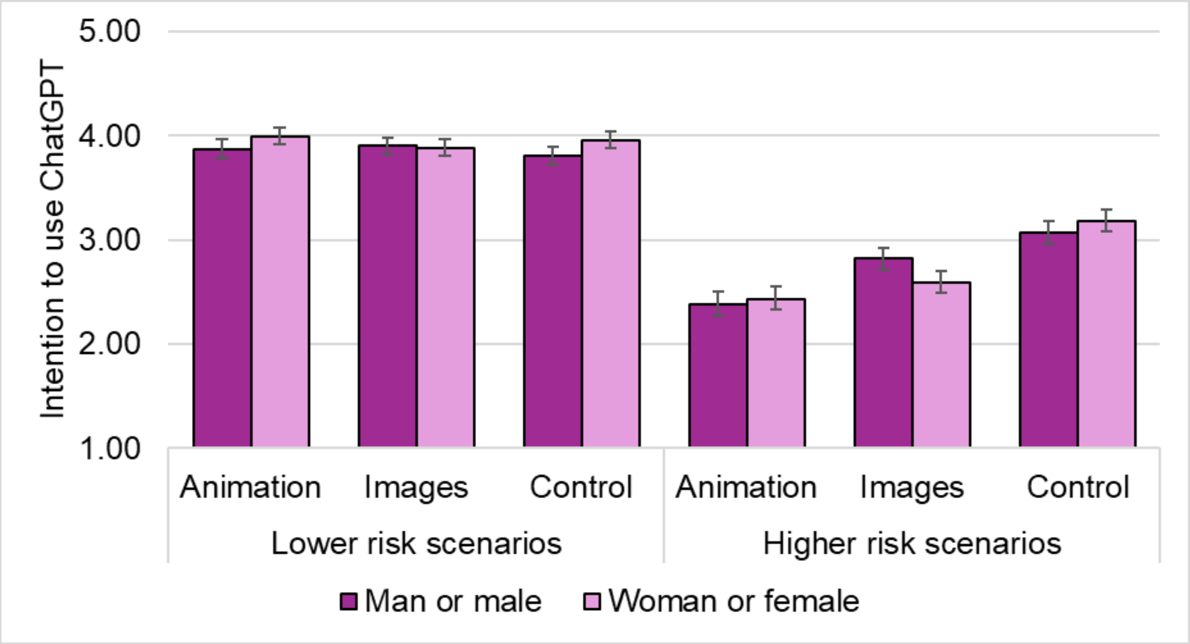

Figure S3. Intention to use ChatGPT, by group, gender and scenario type.
Note: Error bars indicate ±1 SE. Higher scores indicate higher intention of using ChatGPT. The effect of gender compared man or male and woman or female. Figure created using Microsoft Excel.

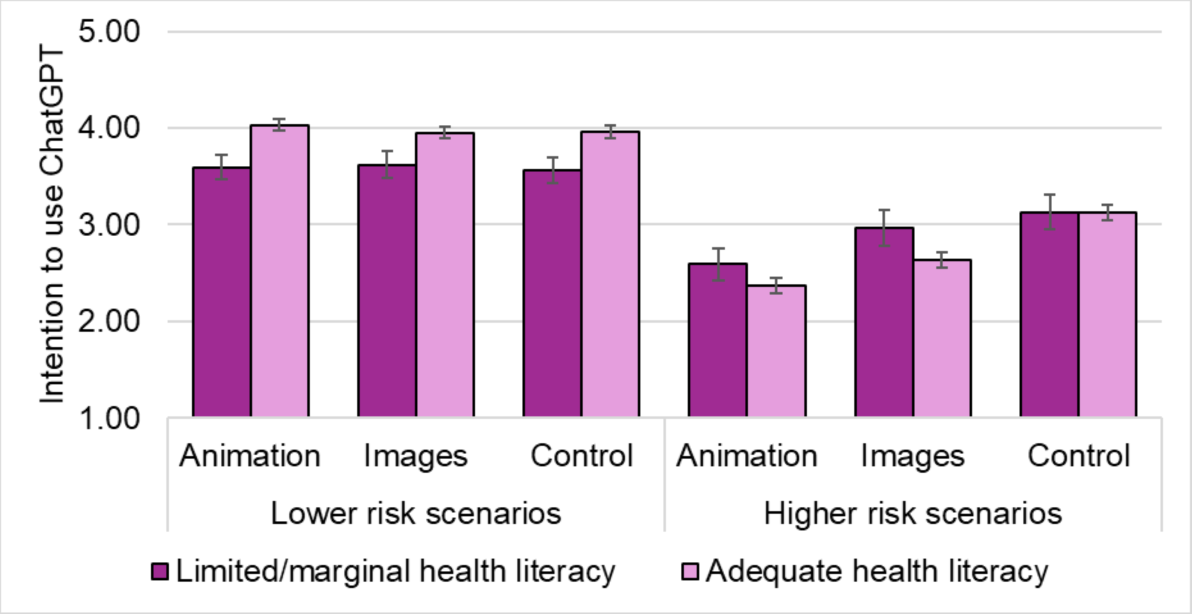

Figure S4. Intention to use ChatGPT, by group, health literacy and scenario type.
Note: Error bars indicate ±1 SE. Higher scores indicate higher intention of using ChatGPT. Health literacy was assessed using the single item screener [11]. Figure created using Microsoft Excel.

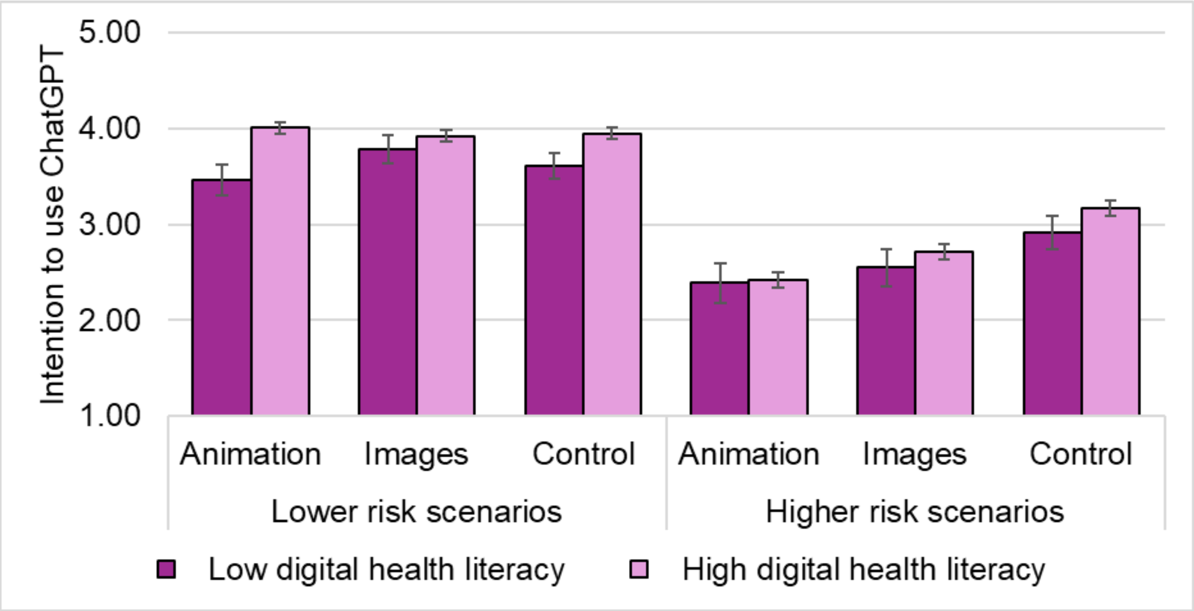

Figure S5. Intention to use ChatGPT, by group, digital health literacy and scenario type.
Note: Error bars indicate ±1 SE. Higher scores indicate higher intention of using ChatGPT. Digital health literacy was assessed using the eHeals [12]. The cut-off for low was based on scores below the midpoint of the scale. Figure created using Microsoft Excel.

References

1. Ayre J, Mac O, McCaffery K, et al. New Frontiers in Health Literacy: Using ChatGPT to Simplify Health Information for People in the Community. J Gen Intern Med 2024;39:573-7.

2. Dunn AG, Shih I, Ayre J, Spallek H. What generative AI means for trust in health communications. J Commun Healthc 2023;16:385-8.

3. Li J, Dada A, Puladi B, Kleesiek J, Egger J. ChatGPT in healthcare: A taxonomy and systematic review. Comput Methods Programs Biomed 2024;245:108013.

4. Wei Q, Yao Z, Cui Y, Wei B, Jin Z, Xu X. Evaluation of ChatGPT-generated medical responses: A systematic review and meta-analysis. J Biomed Inform 2024;151:104620.

5. Sandmann S, Riepenhausen S, Plagwitz L, Varghese J. Systematic analysis of ChatGPT, Google search and Llama 2 for clinical decision support tasks. Nat Commun 2024;15:2050.

6. Haltaufderheide J, Ranisch R. The ethics of ChatGPT in medicine and healthcare: a systematic review on Large Language Models (LLMs). NPJ Digit Med 2024;7:183.

7. Nasra M, Jaffri R, Pavlin-Premrl D, et al. Can artificial intelligence improve patient educational material readability? A systematic review and narrative synthesis. Internal Medicine Journal;n/a.

8. Yeo YH, Samaan JS, Ng WH, et al. Assessing the performance of ChatGPT in answering questions regarding cirrhosis and hepatocellular carcinoma. Clinical and molecular hepatology 2023;29:721-32.

9. Wang L, Chen X, Deng X, et al. Prompt engineering in consistency and reliability with the evidence-based guideline for LLMs. NPJ Digit Med 2024;7:41.

10. Meskó B. Prompt Engineering as an Important Emerging Skill for Medical Professionals: Tutorial. J Med Internet Res 2023;25:e50638.

11. Wallace LS, Rogers ES, Roskos SE, Holiday DB, Weiss BD. Brief report: screening items to identify patients with limited health literacy skills. J Gen Intern Med 2006;21:874-7.

12. Norman CD, Skinner HA. eHealth Literacy: Essential Skills for Consumer Health in a Networked World. J Med Internet Res 2006;8:e9.

Supplementary information C (Survey)

Evaluating social media interventions to support safe use of ChatGPT for health

1. What is this study about?

You may have heard about ChatGPT in the news or on social media. ChatGPT is a publicly available AI chatbot that is free, easy to use and gives quick, human-like responses to user questions and requests. We want to test two interventions to see if they help people use ChatGPT more safely when asking health questions.

2. Who can take part in the study?

You can take part of the study if you live in Australia and are over 18 years of age.

3. What will I be asked to do?

We will ask you to complete an online survey that will ask questions about you, for example, your age, gender and education. We will also ask you to complete some questions about the intervention and how you feel about asking ChatGPT about your health.

We expect this will take you about **15 minutes**.

4. Do I have to take part? Can I change my mind once I've started?
Being in this study is completely voluntary and you do not have to take part. If you decide to take part, you can withdraw any time before you submit the survey. However, once your responses are submitted, they cannot be withdrawn. This is because they are anonymous, and we will not be able to tell which one yours is.

Your decision will not affect your current or future relationship with the researchers or anyone else at The University of Sydney.

To read more information about this part of the study, please download the Participant Information Statement <link to PDF version of PIS>. This study has received Ethics approval from University of Sydney Human Research Ethics Committee (Project No. 2024/255).

I confirm that I have read the Participant Information Statement and consent to take part in this research project as described.

Yes	No

[If ‘yes,’ proceed to survey question on next page, if ‘no,’ survey ends and no data is collected.]

[Demographic questions]

Thank you for taking part in our study. First, we have a few questions about you:

In the last 6 months how often have you used ChatGPT?

Not at all	A few times	Once a month	Once a week	More than once a week

[Screen out participants who have not used ChatGPT at all]

In the last 6 months how often have you used ChatGPT to answer questions about health?

Not at all	A few times	Once a month	Once a week	More than once a week

How old are you?

____

[Screen out participants are less than 18 years]

Do you live in Australia?

- ☐ Yes
- ☐ No [direct to end of survey]

[Screen out participants who do not live in Australia]

What is the highest level of education you have **completed**?

- ☐ Less than Year 12 or equivalent
- ☐ Completed Year 12 or equivalent
- ☐ Trade or technical certificate or diploma
- ☐ University degree
- ☐ Postgraduate/higher degree

[Screen out participants who have respond University degree or postgraduate/higher degree]

What is your gender identity?

- ☐ Female/ Woman
- ☐ Male/ Man
- ☐ Non-binary
- ☐ I use another term (please specify)
  ____
- ☐ Prefer not to say

2) In which country were you born?
   - ☐ Australia
   - ☐ Other (please tell us) ________

[If 'other' answered in previous question] In what year did you move to Australia?

____

What language do you speak at home?

- ☐ English
- ☐ Other (please tell us)

____

Are you of Aboriginal or Torres Strait Islander origin?

- ☐ Yes
- ☐ No
- ☐ Prefer not to say

Which, if any, of the following long-standing health conditions do you have (including age-related conditions)? (select all that apply)

Deafness or severe hearing impairment	A neurological condition (e.g. Alzheimer's, Parkinson's)
Blindness or severe vision impairment	None of these
A longstanding illness (e.g. cancer, HIV, diabetes, chronic heart disease)	
A longstanding physical condition (e.g. arthritis, spinal injury, multiple sclerosis)	
An intellectual disability	

A mental health condition (e.g. depression)	

[health literacy]

The next few questions ask you about your experience understanding health information.*

a) If you need to go to the doctor, clinic, or hospital, how confident are you filling out the medical forms by yourself?

Not at all confident	A little confident	Somewhat confident	Quite confident	Extremely confident

b) How often do you have someone (family member or staff at the clinic or hospital) help you to read health or medical information forms?

Always	Often	Sometimes	Occasionally	Never

c) How often do you have problems learning about your health because of trouble understanding written health information?

Always	Often	Sometimes	Occasionally	Never

d) How often do you have trouble understanding what your doctor, nurse, or pharmacist tells you about your health or about health information?

Always	Often	Sometimes	Occasionally	Never

e) How often do you have trouble remembering instructions from the doctor, nurse, or pharmacist after you get home?

Always	Often	Sometimes	Occasionally	Never

*The first item (a) is a validated single-item heath literacy screener. This was used in the analysis, rather than the full five item scale.

[Digital health literacy]

The next questions will ask about using the internet for health information. For each statement, tell me which response best reflects your experience *right now*. (5-point scale strongly disagree to strongly agree)

1) I know **what** health resources are available on the Internet
2) I know **where** to find helpful health resources on the Internet
3) I know **how** to find helpful health resources on the Internet
4) I know **how to use** the Internet to answer my questions about health

5) I know how to use **the health information** I find on the Internet to help me
6) I have the skills I need to **evaluate** the health resources I find on the Internet
7) I can tell **high quality** health resources from **low quality** health resources on the Internet
8) I feel **confident** in using information from the Internet to make health decisions

In the last 6 months have you used ChatGPT to... (select all that apply)

- ☐ Find out what my symptoms mean (or the symptoms of someone I know)
- ☐ Find out what to do about a specific health issue that I or someone I know has
- ☐ Learn about a specific health condition
- ☐ Learn about healthy lifestyles
- ☐ Help create a plan to improve my health (or the health of someone I know)
- ☐ Learn more about a medicine, test or treatment (e.g. safety, side effects or interactions)
- ☐ Find out if I or someone I know should see a doctor
- ☐ Understand medical terms
- ☐ Interpret results from blood tests or imaging
- ☐ Other (please tell us: ____________)

How much do you trust what ChatGPT says?

Extremely	Quite a bit	Somewhat	A little bit	Not at all

[Randomised to intervention group]

[Image group] In the next section we want you to look at some information about using ChatGPT to answer your health questions. Please look at all 7 images below. There is a timer to allow you to take a closer look at the images. If you finish before the Next button appears, please have another look.

[animation group] In the next section we want you to look at some information about using ChatGPT to answer your health questions. Please watch the whole video. Once you have watched the video the Next button will appear and you can continue the survey.

[control group] In the next section we want you to look at some information about healthy eating. There is a timer to allow you to take a closer look at the image. If you finish before the Next button appears, please have another look.

[post-intervention section]

How much do you trust what ChatGPT says?

Extremely	Quite a bit	Somewhat	A little bit	Not at all

[ChatGPT knowledge]

Please tell us which statements you agree with:

1. It's safe to use ChatGPT in an emergency if you really need to [Yes/**no**]
2. ChatGPT can be useful for answering general health questions [**Yes**/no]
3. If ChatGPT is confident when it tells you how to interpret your blood test results, its answer is more likely to be correct [Yes/**no**]
4. You can trust ChatGPT's answer if it references journal articles that report on clinical trials [Yes/**no**]
5. *It is best to avoid asking ChatGPT for information about a medication's possible side effects. [Yes/no]
6. ChatGPT knows which treatment option is best for you [Yes/**No**]

*On reflection this item wording was unclear and is not included in the analysis.

List four tips that may help make ChatGPT's answers to health questions more accurate*

1: __________

2: __________

3: __________

4: __________

*This variable is not analysed in this manuscript.

[intention to use ChatGPT in health-based scenarios]

Imagine that your uncle has just found out they have gout. The doctor has asked them to get some blood tests done including a test for uric acid. They also mentioned that gout can affect your kidneys. You want to help them find out more about this health condition. You're thinking about using ChatGPT to get you started.

Which of the following questions would you ask ChatGPT ? [definitely avoid | probably avoid | not sure | probably try | definitely try]

1. What is gout?
2. How is gout usually treated?
3. Should my uncle use corticosteroids to treat gout?
4. What does the uric acid test measure?
5. Is it OK if his uric acid test result is 8 mg/dL?

6. You want to learn more about how gout affects the kidneys. You find some information about gout in a well-known medical journal. You think about asking ChatGPT to summarise the information in simple terms.
7. Now imagine that your uncle has been feeling unwell. You're not sure what these new symptoms mean and whether he needs to see a doctor. You type the text below into ChatGPT to help you and your uncle decide:
   For the last few weeks my uncle has had a rash near his eyes. Some parts of the rash have become itchy and have raised red lumps that are getting bigger. It is worse at night than during the day. Does he need to see a doctor about the rash?

[Image/animation groups only] The next section asks you questions about what you thought of the ChatGPT information you saw earlier.

Show how much you agree with the following*:

[7 point scale from Strongly disagree to Strongly agree]
a. I found the information was created personally for me
b. I felt that the information was relevant to me
c. I felt that the information was designed specifically for me

*not analysed in this manuscript

[perceived effectiveness] Please show below how you felt about the information/video you just saw.
The information/video was…

[5 point scale from Strongly disagree to Strongly agree]
a. worth remembering
b. attention-grabbing
c. powerful
d. informative
e. meaningful
f. convincing

[intentions to share on social media]
If you were to see this information/videos online, how likely would you share this information/videos to your socials?

[7 point scale from Very Unlikely to Very Likely]

[perceived usefulness]
Please rate the extent to which you agree or disagree with the following statements:
[5 point scale from Strongly disagree to Strongly agree]

1. The information from this study was new to me.
2. The information gave me useful tips for asking ChatGPT health questions
3. The information helped me think more carefully about which health questions to ask ChatGPT

4. The information helped me think more carefully about how to ask ChatGPT health questions
5. The information helped me think about how to use ChatGPT with other sources of information
6. I intend to apply the information I learnt in this study next time I want to ask ChatGPT a health question.
7. I am confident that I can use this information next time I ask ChatGPT a health question.

Do you have any comments about the information you saw? E.g. How could we improve it?
[free text responses]

Is there anything more that you want to know about using ChatGPT to answer your health questions?
[free text response]

[end of survey – once submitted participants in all groups will receive a link to the two social media interventions]
